# Young Adults and Their Families Living With Mental Illness: Evaluation of the Usefulness of Family-Centered Support Conversations in Community Mental Health care Settings

**DOI:** 10.1177/1074840720964397

**Published:** 2020-10-23

**Authors:** Lisbeth Kjelsrud Aass, Hege Skundberg-Kletthagen, Agneta Schrøder, Øyfrid Larsen Moen

**Affiliations:** 1 Norwegian University of Science and Technology, Gjøvik, Norway; 2 Faculty of Medicine and Health, University Health Care Research Center, Örebro University, Sweden

**Keywords:** young adults, mental illness, family nursing, family intervention, community mental health care

## Abstract

The aim of this study was to evaluate the usefulness of Family-Centered Support Conversations (FCSC) offered in community mental health care in Norway to young adults and their families experiencing mental illness. The FCSC is a family nursing intervention based on the Calgary Family Assessment and Intervention Models and the Illness Beliefs Model and is focused on how family members can be supportive to each other, how to identify strengths and resources of the family, and how to share and reflect on the experiences of everyday life together while living with mental illness. Interviews were conducted with young adults and their family members in Norway who had received the FCSC intervention and were analyzed using phenomenography. Two descriptive categories were identified: “Facilitating the sharing of reflections about everyday life” and “Possibility of change in everyday life.” The family nursing conversations about family structure and function in the context of mental illness allowed families to find new meanings and possibilities in everyday life. Health care professionals can play an important role in facilitating a safe environment for young adults and their families to talk openly about the experience of living with and managing mental illness.

Being diagnosed with mental illness often has a negative impact on many aspects of a young adult’s life including decreased self-esteem, optimism, confidence, as well as difficulties concentrating and carrying out daily taken for granted tasks (McCann et al., 2012). In addition, young adults experiencing mental illness face the developmental challenges of emerging adulthood that include making the transition from living with, to living apart from parents; obtaining education or vocational training; making their way into the workforce; and finding a life partner (Arnett et al., 2014). Being a family member who is caring for a young adult with mental illness can be a highly positive experience through the provision of empathy, love, and support; it may also entail burden and difficulties (Ewertzon, 2015).

## Families Living With Mental Illness

Young adults living with mental illness need support from their family as they strive to find healing and recovery; family members unquestionably play a key role in supporting the young adult’s pathway to recovery (Aass et al., 2020; Lindgren et al., 2015). Parents of young adults describe involvement in informal and professional mental health care as an isolated involvement with lack of being informed, seen, or acknowledged by health professionals (Andershed et al., 2017). Parents and adult children suffering from long-term mental illness describe dependency and influencing each other’s lives. Nevertheless, parents experience being excluded from care, simultaneously being taken for granted and expected to contribute to the care (Johansson et al., 2014). Relatives of inpatients with depression report that health problems, burdens, and worries in everyday life are challenging (Skundberg-Kletthagen et al., 2014). Their lives are often very intertwined with the life of their severely mentally ill family member (Weimand et al., 2010). The well-being of siblings of an individual with a severe mental disorder like psychosis has also been shown to be negatively affected as they experience challenges in relation to be a sibling (Ewertzon et al., 2012).

## Mental Health Care of Families Living With Mental Illness

The range of services for young adults with mental illness in Norway is split among administrative levels. The municipalities have a legal obligation and responsibility for young adults with mental health and mental illness. The regular general practitioner (GP) service in the primary health service plays a key role as a “gate-keeper” of other services and welfare. Community mental health services are staffed by health care professionals such as nurses, social workers, social educators, and occupational therapists. A number of them have supplementary education in mental illness so that they have a preventive role and can offer treatment and follow-up to young adults with mental illness. The importance of involving and acknowledging family as a resource in treatment and care is emphasized (Aass et al., 2020; Schröder et al., 2007; Weimand, 2012) as well as the implementation of strengths-based approaches (Gottlieb, 2013). Studies have found that approximately 18%–34% of young people with high levels of depression or anxiety symptoms seek professional help. Research about this population reports perceived stigma and embarrassment, problems recognizing symptoms, and a preference for self-reliance as barriers for help-seeking (Gulliver et al., 2010). However, insufficient attention has been paid to the care that young adults receive once they are in the health care system (Stroud et al., 2015). Interventions which focus on interactions and the family as a resource for offering unique skills, strengths, resources, and unmet needs are needed. These kinds of interventions may facilitate the experience of mental health treatment and care (Goudreau et al., 2006; Tedeschi & Kilmer, 2005) and increase knowledge and the coping abilities of families (Chesla, 2010). As community-services often rely on the commitment of families and their coping capacity, families should be assessed regularly to ensure that they benefit from the necessary support, education, and provision of resources (World Health Organization, 2013).

## Intervention Studies: Families Living With Mental Illness

The families in this study participated in three Family-Centered Support Conversations (FCSC). The FCSC is theoretical grounded in Wright and Leahey’s Calgary Family Assessment Model (CFAM) and Calgary Family Intervention Model (CFIM) which are strengths-oriented family nursing assessment and intervention models for families living with illness (Shajani & Snell, 2019; Wright & Leahey, 2013). The Illness Beliefs Model (IBM; Wright & Bell, 2009) also guided the FCSC and is based on the principle that it is not necessarily the illness itself, but rather the beliefs about the illness that are potentially the greatest source of individual and family suffering. Emphasis must be placed on recognizing that families, as well as health care professionals, have beliefs that both facilitate and constrain their lives, relationships, behavior, suffering, and healing (Wright & Bell, 2009).

Previous family nursing intervention studies have been conducted with individual and group psycho-educational training, tasks, and therapeutic conversations combined with family interviews involving family members of adolescents and young adults with eating disorders and attention-deficit hyperactivity disorder (ADHD) in a hospital unit. Findings revealed improvement and differences in caregivers’ emotional and cognitive support, illness beliefs, emotional functioning, caregiving demands and caregivers, and patient behavioral difficulties (Gísladóttir et al., 2017) and better quality of life and social functioning for caregivers (Gísladóttir & Svavarsdóttir, 2017). Intervention studies with patients and family members in acute psychiatric hospital units, who received family nursing conversations focused on family strengths, reported that family members perceived significant higher emotional and cognitive support after the intervention (Sveinbjarnardóttir et al., 2013). In addition, benefits were observed in families of young people living with severe mental illness regarding revisiting and building new connections among family members, and strengthening and supporting the family network (Sveinbjarnardóttir & Svavarsdóttir, 2019).These family nursing intervention studies were guided by the CFAM and CFIM and/or the IBM as the theoretical framework to inform the interventions offered (Gísladóttir et al., 2017; Gísladóttir & Svavarsdóttir, 2017; Svavarsdóttir et al., 2019; Sveinbjarnardóttir et al., 2013; Sveinbjarnardóttir & Svavarsdóttir, 2019). Nevertheless, to our knowledge, strengths-oriented family support conversation studies with young adults and their family as the unit of care in community mental health services have only been reported to a limited extent. Moreover, interventions need to be explicitly tested in young adults because they can be influenced by many factors includingthechallengesofemergingadulthood, maneuvering developmental transitions, and adjusting to adult mental health care (Lindgren et al., 2015). Therefore, knowledge from this study can provide direction about how to meet young adults and the family’s needs and will also help to expand information about family focused care for this population of families nationally and internationally.

## Aim of the Study

The aim of this study is to explore and evaluate how young adults living with mental illness and their families experienced a family intervention called FCSC.

## Method

This intervention study used an explorative qualitative design with a phenomenographic approach (Marton, 1988). Phenomenography was chosen to incorporate variety and differences in how the phenomenon was experienced, conceived, and captured at the family level. In this study, we endeavored to capture data at the family level to identify multiple perspectives and focus on the family as a unit. Phenomenographytakesasecond-orderperspective, meaning that it offers different ways of conceiving the phenomenon that are of interest and how it is described (Marton & Booth, 1997). In this study, the phenomena was the family members’ experiences of receiving the FCSC intervention.

### Recruitment of Participants

The focus of this study was on families living with mental illness of a young adult family member where family was a self-identified group of two or more individuals who were or were not related by legal or blood relationships and who functioned in such a way that they considered themselves to be a family (Whall, 1986). Health care professionals recruited patients who were young adults living with mental illness in urban and rural communities and asked them both verbally and in writing to participate, both in the family conversation and in a follow-up family research interview. Family members (one or two) were recruited through the young adult patient who asked his or her family members to participate in the study. Family members then gave the patient permission to submit their name and telephone number to health care professionals. Both the patient and the family members gave oral and written consent (International Committee of Medical Journal Editors, 2018).

#### Patient inclusion and exclusion criteria

Inclusion criteria for patients: aged 18−25 years, facing mental illness and strain, impaired function associated with distress, symptoms, and diagnosable mental disorders; living alone or with family and/or friends and/or others; could speak and read Norwegian; and had contact with community health services related to mental illness. Exclusion criteria for patients included cognitive impairment; psychotic state; active alcohol or drug abuse; or living in a residential home for persons suffering from mental illness.

#### Family member inclusion and exclusion criteria

Family members over 18 years old who were defined by the young adult to be part of the family, and who were able to speak and read Norwegian. This study excluded family members who showed evidence of cognitive impairment, psychosis, or active alcohol or drug abuse.

### Description of the Intervention: FCSC

FCSC included the young adult who was suffering from illness, those designated as belonging to his or her family, and a mental health care professional. Operationalized within a non-hierarchical therapeutic relationship, three conversations with each family were conducted by the same mental health care professionals that include a psychiatric nurse, social worker, and social educator with advanced training in mental health. The goals of the FCSC were to (a) shift the focus from a deficit- or dysfunction-based family assessment to a strengths- and resource-based family conversation including those persons who were important in the patient’s life and (b) recognize that family members serve a variety of roles including advocate, care provider, trusted companion, and surrogate decision maker (Levine & Zuckerman, 1999; Wright & Leahey, 2013). The mental health care professionals involved in this study had completed a 2-day educational program on family assessment and intervention as well as skills training with different clinical vignettes from mental illness care (Benzeinetal.,2012; Sveinbjarnardóttir et al., 2011; Wright & Bell, 2009; Wright & Leahey, 2013).

#### Procedure for FCSC

##### First session

Each family member was invited to relate their narrative about their experiences and beliefs in relation to everyday life. Family structure, development, and function were explored and assessed to later reflect on these aspects of family functioning, and the strengths and resources that can have an impact on everyday family life (Benzein et al., 2015; Gísladóttir & Svavarsdóttir, 2011; Wright & Leahey, 2013).

##### Second session

The focus of the second session was on cognitive, affective, and behavioral domains of family functioning and strengths and resources within and outside the family. The impact of problems/illness on the family was assessed. Problem-solving skills, coping strategies, and strengths were elicited, and change invited. Aspects of family functioning, strengths, and resources within and outside the family were reflected on.

##### Third session

The focus of the third session was on families’ experience of everyday life and support strategies for the future. Families were commended for their and individual strengths, competencies, and resources. While three conversations are recommended, the health care professional must evaluate if families need more than three conversations (Benzein et al., 2008, 2012). If families needed additional support conversations, they were free to contact the mental health care professionals.

### Data Collection

Family research interviews with open-ended questions were conducted with each of the seven families who received the FCSC intervention. Data were collected 1 to 2 months after the FCSC was completed. The families were allowed to narrate freely about their experience of the FCSC. The initial research interview question asked was: “You have participated in three FCSC. How did you experience the FCSC?” The focus was on how the conversations were experienced and what was experienced. To gain a deeper sense of the experiences, follow-up questions were asked such as: how? who? can you tell more? what do you mean? is it always so? to elicit a richer and more detailed description, and further questions were adjusted to the participants’ response. In each family research interview, consideration was given to the developmental level of the patient (Donalek, 2009) who was asked first to talk about his or her experiences in a relaxed and accepting atmosphere, giving the patient time and space to answer questions, and focusing the conversation in the direction of the phenomenon (Lepp & Ringsberg, 2002). When the patient had no more to say or was reluctant to answer, the family members were asked the same questions. Throughout the family research interview, the dialogue alternated between the family and the interviewer asking questions. The family research interviews were conducted by the first author and took place either at the family’s home, at the mental health care service office, or the university according to the wishes of the participants. Notably, one patient and a partner who participated in the FCSC did not participate in the family research interview due to private concerns. The interviews lasted 50−65 min and were audio-taped and transcribed verbatim by the first author.

### Ethical Considerations

Ethical considerations and guidelines with respect to confidentiality, integrity, and the voluntary participation of the participants were followed throughout the study (International Committee of Medical Journal Editors, 2018; World Medical Association, 2001). Both the patient and family members received written and oral information and gave their written consent. They were informed that the material would be treated confidentially. The patient gave written informed consent for the publication of patient information (International Committee of Medical Journal Editors, 2018). The Regional Committee for Medical & Health Research Ethics (REC) found the Research Project, Ref: 2017/717, to be outside the remit of the Norwegian Act on Medical and Health Research) and the project could therefore be implemented without its approval. Approval was given by the Data Protection Official for Research (NSD), June 2017, Ref: 54696.

### Data Analysis

The data were analyzed as a “pool of meanings” (Marton & Booth, 1997) inspired by Dahlgren and Fallsbergs’ (1991) steps for analyzing phenomenography studies: (a) Familiarization—the researcher read through the transcripts to become familiar with all the details and establish an overall impression of the data; (b) Condensation—the most significant statements made by the families concerning the phenomenon were condensed to give a short but representative version of the entire dialog; (c) Comparison—significant conceptions were compared to find sources of variation and agreements in how the phenomenon were experienced; (d) Grouping—concepts appearing to be similar were grouped together; (e) Articulating—a preliminary attempt was made to describe the essence of the similarity within each group of concepts; (f) Labeling*—*descriptive categories were labeled based on findings of suitable linguistic expressions; and (g) Contrasting—description categories were compared to ensure that each category was mutually exclusive and at the same level. The last three steps were repeated several times. The outcome space refers to a horizontal structure in which the descriptive categories reflect the distinctions of the FCSC.

## Findings

### Research Participants

A sample of 19 family members from seven families participated in the FCSC with mental health care professionals in four different municipalities in Norway in the period from December 2017–May 2018. Out of the 19 family members, 17 consented to participate in family research interviews. The sample represented variation with respect to age, gender, education, and occupation (see Table 1). Mental illness among the young adults ranged from depression and anxiety distress or disorders, personality disorders, ADHD, and eating disorders.

**Table 1.** Participating Young Adults and Family Members.

Characteristics	Patients	Family members
**Age (years)**	19−23	20−55
**Gender**		
**Male**	1	5
**Female**	5	6
**Relationships**		
**Daughter**	5	
**Son**	1	
**Spouse**		1
**Mother**		6
**Father/stepfather**		4
**Educational level**		
**Secondary school**	2	
**High school**	3	7
**Apprentice**	1	
**University**		4
**Occupation**		
**Work**	1	7
**Job seeker**	1	
**Sick leave**	1	1
**Disability benefit**		1
**Work assessment allowance**	1	
**Other**	2	2

### Families’ Experience of the FCSC

The findings describe the families’ experiences of FCSC under two descriptive categories, “Facilitating sharing reflections on everyday life” and “Possibility of change in everyday life.” The descriptive categories embody five concepts that comprise the outcome space (Marton & Booth, 1997) (Table 2).

**Table 2.** Experiences of the Usefulness of the Family-Centered Support Conversations: The Perspectives of Young Adults and Their Families Living With Mental Illness.

Descriptive categories	“Facilitating sharing reflections on everyday life”			“Possibility of change in everyday life”	
Concepts	The unfamiliar conversations	A team with mutual understanding	Experiencing a change in the patient approach	Awareness of strengths and resources	Support in everyday life on regular basis

#### Facilitating sharing reflections on everyday life

The FCSC facilitated an opportunity to share and reflect on the family’s beliefs on the past, present, and future of everyday family life related to symptoms, problems, challenges, worries, and hopes. This descriptive category includes three concepts: the unfamiliar conversations, a team with mutual understanding, and experiencing a change in the patient approach. The category describes the FCSC in regard to potential benefit for individuals and family, state of consciousness, and degree of confidence after reflecting on everyday family life together with a mental health care professional.

##### The unfamiliar conversations

How the families experienced the FCSC varied, and for most, it was a new experience, perceived as strange, unpleasant, and uncomfortable but also positive, beneficial, and safe. The patients, who were used to having conversations with mental health care professionals alone, described the FCSC as strange and unpleasant. One patient said, “It’s a bit strange because I don’t usually talk about these things with my mother.” Some of the patient’s symptoms, strain, and difficult thoughts had never been shared with the family prior to the FCSC because it was too hard to talk about. However, when struggling to explain, reveal, and talk with the family about difficult topics such as how mental illness, decreased confidence, and self-esteem affected them in everyday life, the patients were reassured by the presence of the mental health care professional. A patient said, “It’s been almost taboo. I’ve hardly ever wanted to talk about it. So, it was good that she (health care professional) was in charge, then we talked about it.” Other families stated that after each session they talked about how good they felt about the conversations.

The topics of discussion varied within the three conversations and experienced by some as being defined by others or occurring randomly. They talked about how the situation was at the moment, how things had been and what thoughts they had about the future. Sharing thoughts was described as useful and informative, and dwelling on each other’s experience enabled them to see the situation from other angles. One father said, “We realized that we maybe hadn’t understood how bad it really is for A . . . We found out how difficult it is for her through the conversations with the health care professional.” Listening to the patient’s description of everyday life was experienced as painful and surprising, and as a moment of new realization. Prior to the FCSC, they did not know how seriously the patients were affected by mental illness, and what the patient needed in situations that trigger severe symptoms. A father said,

It was much worse than we thought. A bit surprising but good to hear. If the family is going to the store, for example, and she is coming along, she has to be prepared. It can take half an hour till she’s ready to leave. We didn’t understand why she didn’t come in, and we nagged a lot sometimes. We weren’t aware that she had to have this time for preparation . . .

However, for some family members, the FCSC did not lead to new understanding but was a repetition of what they had been through because they had discussed the topics in the conversations beforehand and dealt with them thoroughly. Others described having to “speak loudly” in a sharp tone to make their opinions on how to interact with their patient heard. The justification for this was that they live with them and know what works and does not work concerning symptoms and behavior in everyday life related to the patient’s mental illness.

Topics that family members earlier did not dare to bring up or mention to the patient because they were afraid of stepping too close were naturally brought up and talked about in the FCSC. One mother talked about how she experienced that the patient excluded her from information. She did not know anything about the mental health status or the care process, which led to self-interpretation and conclusions based on assumptions and worrying.

The patients were familiar with most of what the family members shared in the conversations, such as concerns regarding the patient being suicidal, having severe anxiety and being depressed, as well as concerns about education, getting to work, and even keeping a job.

##### A team with mutual understanding

Meetings concerning the patient’s mental health issues with the general practitioner physician, therapist, and social security officer at the Labor and Welfare Service were familiar. However, the families described attending few conversations with focus on family strengths, resources, and support with mental health professionals prior to the FCSC. They described being able to speak honestly and sincerely about emotions and thoughts regarding situations in the past concerning family everyday life for the first time. One mother shared how she felt when the patient had been seriously ill and hospitalized and excluded the family from being informed. The families sorely desired collaboration with the health care professionals, with the aim of working in the same direction. Still they experienced the opposite. A mother said,

We as a family and the help we give A works to a certain extent if the team around us work in the same way. However, when the team don`t work with us but against us, they actually do a lot more damage.

Family members emphasized that they sense when symptoms worsen, but the patients often deny or answer their concerns dishonestly. They do not have to share everything but struggling must be talked about to be able to be supportive. According to one mother, “. . . I ask and then ‘Yes, yes, I`m alright,’ but maybe it’s not alright. I can see it, but each time I ask, she replies, ‘I’m tired’.” A patient said, “I don’t like people knowing too much about me. I want to deal with things myself, not be seen as different.” Getting a third person’s view was helpful in relation to how the patient’s mental illness affects the management of daily activities such as getting to work. A patient replied, “Yes, I also found it a bit reassuring because NN (mental health care professional) could explain to my mother and boyfriend how things were. I’m not always good at explaining and saying things.” The intervention made it possible for family members to learn how they can approve, support, and help, thereby enabling the family members to enlighten each other and mental health care professionals on what works when living with mental illness. One mother experienced becoming aware of how to communicate so that misunderstandings do not occur, and the importance of giving positive feedback and praise. One stepfather said, “He has apparently not appreciated boundaries in relation to us . . . but in one of this meetings he admitted he actually appreciated some boundaries.”

The patients described being absent minded or distracted and perceiving no more than one third of what was going on in conversations and not always being able to explain and express needs. Therefore, it was important to have family members present to help grasp what was said. Others stated that they did not pay attention when family members spoke to health care professionals. Notably, not having all the family present in conversations was seen as a drawback. A mother said, “No, if it’s going to be of any use, I think the four of us have to talk together. That is, if we as a family are to benefit from it”.

##### Experiencing a change in the patient approach

The families described mental health care professionals asking several questions concerning how the families were coping at home. In addition to being concerned about the patient, mental health care professionals were sincerely concerned about family members’ time for each other and for taking care of their own relationships. The mental health care professionals described bringing forth strengths and resources within the family through mapping and reflecting on actions and activities they perform in everyday life. One mother said, “He wondered about what you do when things are going well, kind of . . .? What did you do then? What happened that day? And what is good about those days? Both NN (patient) and we were asked that question.”

Mental health care professionals were described as seeking approval from the patients regarding the sharing of information although the latter had given them oral and written consent to access family members’ insights into health information. A father said,

The so-called data protection in psychiatry is a drawback. If NN (patient) had told that I am not supposed to know things, that would be ok for me. That’s something else, but NN (patient) wants to be open and wants us parents to help him, but still mental health care professionals hold back.

Family members said that they urged the mental health care professional to listen to them. Thus, the FCSC had some significance in that health care professionals gained greater understanding through listening to the family describing how they were functioning at home.

The families perceived that mental health care professional focused on statements the family disagreed on and asked for clarification before they moved on. The mental health care professionals commenced the dialogs with a couple of questions and did not interrupt if the conversation flowed freely, so the families experienced the topics as a bit random. Others experienced setting goals from the first, second, and third conversation, so that it was not random in that there was an overall emphasis on positive activities, that is, a focus on things they did well. A mother reported, “We kind of agreed on setting some goals from one meeting to the next. That we should try out whatever made things better.” The health care professionals gave information on health care support and actions aiming to unburden the family as well as suggestions as to how families could solve problems in everyday life, which was perceived as positive. A mother said, “I do know we talked about remedial actions for A. What NN (health care professional) could help us with as regards applying for respite care in order to give us some relief.”

### Possibility of Change in Everyday Life

This descriptive category includes two concepts: awareness of strengths and resources and support in everyday life on regular basis. Mapping the family as a whole facilitated the development of new meanings and possibilities and unwrapped strengths, competence, and skills. In addition, it promoted preparedness to deal with everyday life and put in place support strategies for the future.

#### Awareness of strengths and resources

Mapping the family roles, interaction and functioning within the families was experienced as valuable. In practical terms, this meant assessing who is in the family and how those individuals’ function within the family. Some families developed a diagram of the two-generation family genogram (family tree), while others did the family mapping through spoken dialogue. The family mapping revealed how the patients position themselves and other family members. A patient said, “I put myself as far out as possible because I don’t want to be in the way,” and the father replied, “. . . NN drew me as the core” . . . then he put himself as far out as possible on the tip of a branch . . .”

The family mapping was experienced as a moment of realization because the family members had not previously considered the connections between themselves in that way. Mapping was experienced as giving both the family and mental health care professional’s insight and the opportunity to become acquainted with or conscious of unspoken realities.

Regardless of whether it entailed drawing the family tree or just talking about who is in the family and how they function, family mapping was described as beneficial due to the family becoming more aware of what they meant to each other and did together, because when things went wrong, the feeling of remorse often cast a shadow over everything. A mother said, “It’s pretty useful to go over it and see that we actually do positive things too, which is good.” Awareness of interactions within the family and own reactions when things went wrong or there was a conflict was the subject of discussion, and importantly, this had rarely been discussed before. One mother described becoming aware of whether they paid each other respect or took each other into account.

Families described doing the best they could based on their assumptions. They learned to emphasize the importance of giving the patients approval for things they achieved rather than focus on problems when they did not cope. Others knew already that the whole family functioned as a team with strengths and resources.

##### Support in everyday life on regular basis

The patients found it easier to speak exclusively with mental health care professionals who were involved in their care. They reported that mental health care professionals viewed things differently than family members who saw them daily. Yet family involvement in care was worth having. A patient stated, “Think there should be more conversations. As mentioned, in everyday life the effect fades away after a while. No, I feel as if there’s not much talk of it anymore. Things return to the usual, old routines.” Further contact with mental health care professionals was also desirable for family members, who missed having someone to talk to when they came up short in supporting their ill family member. Family members described being in need of guidance on issues concerning themselves being at work, worrying about the patient who was home alone—potentially suicidal and not getting to work. Others felt it was up to the patient to decide together with the mental health care professional when family should take part in future conversations, despite the fact that the mental health care professional had told family members to get in touch when they wanted another meeting.

Family members expressed a need for the patient to live close to the family because it provided a feeling of being safe and supported. However, mental health care professionals emphasized the importance of the patient being as independent as possible to tackle everyday life in the future with extra support and help from the health care services in daily activities. Both the mental health care professionals and the family felt that progress had been made during the FCSC. The families described patients as being in a better state of mental health and function level now than 2−3 years ago. A mother stated, “I feel you have come quite a long way, that you are in better health now. In any case, we have a completely different starting point now.” Nevertheless, the families stressed the value of mental health care professionals asking if they could contribute with help and support, even though the families did not necessarily need this.

## Discussion

The aim of this study was to explore how young adult patients living with mental illness and their families experienced the FCSC. Findings highlighted the families’ desire to be included in mental health care through FCSC that focused on how to be supportive and acknowledged family strengths and resources. Sharing beliefs about everyday life and assessing and reflecting on family function and structure facilitated new meanings and possibilities in everyday life. Mental health care professionals play an important role in facilitating a safe environment for sharing in a non-hierarchical and co-creating relationship.

### *Facilitating Shared Reflections* on Everyday Life

The findings of this study highlighted that on the one hand that patients wanted to include their family in the FCSC to achieve increased understanding, while still dealing with things on their own; on the other hand, they feared to be looked upon differently, resulting in their concealment of certain aspects of mental illness in daily living. Significantly, it was challenging for the patients to talk to family about difficult and taboo topics such as how mental illness affected them—at school, at work, and in social life—and their decreased confidence and self-esteem. However, with mental health care professionals by their side, the patients in this study felt more confident about including family members and disclosing their problems. Moen et al. (2014) similarly describe family members valuing a neutral third person leading the discussion and ensuring that they kept to the subject. This indicates that mental health care professionals build trustful relationships with the patient and family members, framing family support conversations as a safe arena. Sveinbjarnardóttir and Svavarsdóttir (2019) assert that mental health care professionals who have the knowledge, training, and capacity to build a partnership with the family as the unit of care can improve patient services.

According to Woodgate et al. (2017), disclosure or non-disclosure is often grounded in the fear of being stigmatized, treated differently, and/or fear of being rejected by their family. Even though it was hard, disclosure enabled understanding and acceptance from the family and facilitated a dialogue about how to best help and support the patient. The findings of Schröder et al. (2006) indicate that de-dramatizing mental illness reduces or avoids stigmatization of the person with mental illness. On the one hand, disclosure made family members realize shortcomings in their own understanding due to exclusion or concealment. This was also painful because they became aware of how much the patient was struggling to manage everyday life. On the other hand, family members had the opportunity to get answers to issues they did not know about or to questions they had not dared to ask earlier. Stengård and Appelqvist-Schmidlechner (2010) and Woodgate et al. (2017) similarly describe how patients living with mental illness often seem to underestimate the need for help from others and try to deal with their problems on their own, experiencing difficulty communicating their thoughts at times. They expressed the need for additional pathways to share how they feel.

In Norway, the age of majority is 18 years and, from that point on, an individual can decide whether family members are to be given information regarding one’s health issues (Ministry of Health and Care Services, 1999). Family members in this study emphasized that the patient does not have to tell them everything, but it is important to share symptoms, status of illness and suffering, as it enables them to give help and support. Similarly, Andershed et al. (2017) found parents’ need to know about symptoms, the illness, the illness trajectory, and treatment and how to read and understand signs of suffering.

How health care professionals relate and listen has implications for families and matters to them (Wright & Leahey, 2013). Families in this study experienced that mental health care professionals were concerned about how the families were doing at home, whether family members had time for each other and took care of their relationships as well as the patient’s health well-being. This is in contrast to Weimand and colleagues (2011) who found family members reported not being seen, listened to, or understood in mental health and psychiatric in- and outpatient care. Interpersonal relationships and good communication between health care professionals, patient, and family members are key factors in quality of care from the perspective of family members (Schröder et al., 2007). According to Freire (2018), families and health care professionals who encounter each other in dialogic relationships are equally partners, aiming for mutual understanding and finding new words to describe reality, thus enabling change. A dialogical approach is related to and dependent on humility, faith in humans, trust, hope, and critical thinking. According to the CFAM (Wright & Leahey, 2013), the relationship between the mental health care professionals and families is characterized as non-hierarchical and a co-creating of reality. Nevertheless, this study supported the earlier findings of Ewertzon et al. (2010) that cooperation between staff and family members was problematic in the sense that families experienced that their opinion on how to best interact with the patient was not valued. To build a family–nurse relationship, Wright and Leahey (2013) recommend it is useful for health care professionals to reflect on his or her contribution to the therapeutic relationship before meeting with the family and, at the end of the meeting, to invite the family’s reflections about the family–nurse relationship.

A growing body of literature emphasizes the importance of exploring beliefs that shape individual and family narratives of everyday life when living with illness and coping strategies within families (Bell & Wright, 2011; Wright & Bell, 2009). Personal narratives and reflections are significant and joined closely together and are intended to facilitate the appearance of new beliefs and the discovery of alternatives or new meanings that can have an impact (Benzein et al., 2015). Findings showed that beliefs appeared in the family’s narratives, evolving through their history together as a family and revealed when others could confirm and give further examples. Similar to Wright and Bell (2009), we found that understanding beliefs develops in interaction with others over time; however, beliefs are not static and evolve like nurses and families do. In addition, the revealing of new beliefs made acceptance for mutual realities possible and increased understanding for each other’s perspectives on everyday life. This was significant, not only for the families but also for the mental health care professionals. Benzein et al. (2008) suggests that health care professionals’ task is to listen to what the families really says and not what the health care professionals think the families says or means. In this way, health care professionals can take a participatory position in the conversation rather than an influencing one. Families in this study reported that their own beliefs were respected and viewed as equally legitimate by mental health care professionals. However, some experienced that mental health care professionals were more concerned about protecting the patient’s legal rights regarding confidentiality. According to Weimand and colleagues (2013), the trusting alliance between nurse and patient is governed by confidentiality and respect for the patients’ autonomy (Dreyer & Strom, 2019). Mental health care professionals were perceived to be continuously seeking approval from the patients regarding sharing information, despite the latter having given consent. This indicates that mental health care professionals are afraid of acting illegally (Weimand et al., 2013), or misunderstand the law of confidentiality, resulting in barriers for collaboration between families and professionals (Aass et al., 2020; Solomon et al., 2012). Notably, the FCSC were significant in the sense that mental health care professionals showed increased understanding of family everyday life after listening to the family’s narratives. Confidentiality protects the patient but can also be viewed as an obstacle preventing relatives receiving information and participating in care (Schröder et al., 2007). Even though confidentiality makes it difficult for mental health care professionals to talk to family members, there is some scope within statutory regulations that enables a kind of transparency and does not prohibit health care professionals from listening to the family (Weimand et al., 2011).

### Possibilities of Change in Everyday Life

Findings revealed that assessing family roles, interaction, and function either through visualization of a family genogram or through a spoken dialogue facilitated the process of self-reflection and opened up for a new understanding and awareness of what family members meant to each other. According to Wright and Bell (2009), the process of self-reflection is essential to the co-evolution of new, more facilitating beliefs, meanings, and opportunities. In this study, the use of a structural assessment tool like a genogram seemed to have significance for visualizing and facilitating reflections of family positions and hierarchy, who participates in everyday life, and how communication, emotions, and interactions influenced family behaviors. Wright and Leahey (2013) state that the purpose of the genogram is to describe and understand family members’ relationships with each other; however, the visual impact of the genogram on families seems to concretize these family connections in a new and meaningful way. The families in this study described family deficits, strengths, and resources and became more aware of not only how they interact and activities they do together but also what they meant to each other. When health care professionals focused on strengths, the families reported that it was good to hear that they were doing something right. Gottlieb (2013) describes a focus on strengths as helping families to see themselves in a new light. When health care professionals adopt a salutogenic approach, opportunities and resources rather than deficits are emphasized (Benzein & Saveman, 2008; Langeland, 2014). Nevertheless, mental health care programs for youths often tend to focus on what is going wrong in families rather than what is going right and fail to see and appreciate the family’s strengths and competencies. In addition, families are perceived as lacking ability to solve problems or cope without the help of the professional (Gottlieb, 2013). According to Stengård and Appelqvist-Schmidlechner (2010), this can have the potential of stigmatizing, undermining motivation, or discouraging young people from becoming involved in support programs.

The families agreed on the importance of proceeding with support-conversations with mental health care professionals as an arena for continuity of sharing and guidance. However, they did not agree on who should decide when and how often the conversations should occur. The patient and the mental health care professionals wanted family members to initiate meetings, while family members believed it was up to the patient, together with the mental health care professionals, to decide. Community services often rely on family commitment, support-capacity, and competencies (World Health Organization, 2013). Consequently, a family–mental health care professional relationship should include and be characterized by family and mental health care professionals each bringing expertise, strengths, and resources to the relationship and the valuing of reciprocal and non-hierarchical relationships where each person’s contribution is acknowledged and valued (Aass et al., 2020; LeGrow & Rossen, 2005).

Although the FCSC was intended to influence support in family everyday life, the specific outcome can never be predicted in advance. Families carry out their functions through their subsystems (Wright & Leahey, 2013), and findings in this study revealed that the inclusion of many family members was needed if the FCSC was to make an impact on everyday life. According to Wright and Leahey (2013), to be effective, there must be a fit between the intervention offered and the structure of the family. When fit is absent, there is a possibility of no effect. The families decided who participated in the FCSC and this varied across different persons at various times. Handling this with flexibility and adjusting to the family’s situation is recommended (Benzein et al., 2012). This underscores the importance of health care professionals assessing family functioning and structure and asking who is in the family and whether others should be invited (Wright & Leahey, 2013). Mental health care professionals should ensure a specific focus on family structure because it is insufficient to focus on a family everyday life situation with problem solving when the specific family structure is unknown (Wright & Leahey, 2013; Svavarsdottir & Gisladottir, 2019).

In this study, the four criteria offered by Guba (1981) were used to ensure trustworthiness. *Credibility* was strengthened by the sample of seven families ensuring variation in different ways of experiencing the phenomenon (Marton & Booth, 1997) and by the broad sample in terms of different family relationships, gender, and age, which gave rich descriptions (Sandelowski, 1986). *Dependability* was ensured by asking the families the same open-ended questions and gave the participants the opportunity to contribute experiences if they had not been covered during research interview. The first author carried out all the research interviews. To strengthen trustworthiness and authenticity, the authors have reported the findings with quotes from both patients and family members. *Confirmability* was enabled by establishing an “audit trail” (Guba, 1981) describing all steps in the analysis process. Analyzingfamilyresearchinterviewsinthis phenomenographic study was challenging because the experience of the therapeutic conversations was shared across multiple families. However, the involvement of all researchers in the analysis process was a strength. *Transferability*: We believe this study contributes relevant knowledge that may apply to other families within similar contexts. One possible weakness may be the risk of the interview reflecting the experiences of individuals rather than the family as a whole. However, data on both individual and family level were generated by focusing on the family as a unit. The family members in the study are distinct individuals yet they share a common history, strengths, and belief systems and have close contact with one another. Collecting data on a family level increases knowledge and the discovering of the shared family experiences and family meaning that emerge with an illness experience (Chesla, 1995; Eggenberger & Nelms, 2007). The assumption is that family experiences of the FCSC are the sum of the subjective views of each individual family member (Åstedt-Kurki et al., 2001). The families spoke openly in the interview situation and were willing to share their experiences with each other, even if their stories included new thoughts that the family had never discussed. Participating in family research interviews can be a beneficial learning experience for the family as they become more aware of each other’s beliefs and opinions. One limitation, however, could be if family members are not able or willing to be open and share their experiences with each other (Eggenberger & Nelms, 2007; Moen et al., 2014).

The research interviews included issues that were sensitive for the families. As a psychiatric nurse, the interviewer (first author) was skilled in talking to patients with mental illness and their families and was aware of the power balance in the interview (Marton & Booth, 1997).

Family conversations like the FCSC, which is theory-driven (Wright & Bell, 2009; Wright & Leahey, 2013), have a major strength in that they are based on research and previously tested, although to our knowledge not with young adults living with mental illness and their family. The FCSC in this study were examined in real-world community mental health settings and contribute to knowledge about family nursing intervention with this unique population of families.

## Conclusion

This study extends knowledge about the usefulness of FCSC for families and their young adult child living with mental illness. From the participating families’ perspective, there is a desire to be included in mental health care through FCSC focusing on how to be supportive and identify family strengths and resources. One way to meet this need is to offer FCSC as a central complement to usual mental health care practice. The family participants benefit from listening to and reflecting on each other`s beliefs about their daily life and their family functioning. Mental health care professionals play an important role in facilitating a safe environment for sharing.

To acknowledge that mental illness is a family affair and thus focus on the family as the unit of care requires a conceptual shift, even a paradigm shift, by health care providers. Clinicians require specific knowledge and skills to enter into these important conversations with families living with mental illness. Resources such as the International Family Nursing Association (IFNA) Position Statements on Generalist Practice (IFNA, 2015) and Advanced Practice (IFNA, 2017) with families may be useful to guide the development of knowledge and skills necessary for family-centered practice. Further exploration is needed about the family beliefs about mental illness and how the family perceives support and the quality of care offered to them. Finally, this study should be followed by further research evaluating FCSC intervention with a larger sample size of families, with the inclusion of a comparison group, and conducting more mixed methods studies about the benefits of bringing the family together for family-centered conversations in the context of mental illness.
